# Comparative Evaluation of Clinical Outcomes Following Endovascular and Hybrid Repair of Aortic Arch Aneurysms

**DOI:** 10.3390/jcdd13080396

**Published:** 2026-08-18

**Authors:** Yulia Panteleeva, Almaz Vanyurkin, Ekaterina Verkhovskaya, Sergey Kogay, Natalya Maystrenko, Mikhail Chernyavskiy, Dmitry Kudlay, Anna Starshinova

**Affiliations:** 1 Almazov National Medical Research Centre, 197341 St. Petersburg, Russia; 2 Department of Pharmacology, Institute of Pharmacy, I.M. Sechenov First Moscow State Medical University, 119991 Moscow, Russia; 3 Institute of Immunology FMBA of Russia, 115522 Moscow, Russia; 4 Faculty of Bioengineering and Bioinformatics, Lomonosov Moscow State University, 119991 Moscow, Russia; 5 Department of Mathematics and Computer Science, St. Petersburg State University, 199034 St. Petersburg, Russia

**Keywords:** aortic arch aneurysm, in situ fenestration, aortic arch endovascular repair, hybrid treatment

## Abstract

Objective: The aim of this study was to evaluate the efficacy and safety of endovascular and hybrid treatment approaches in patients with aortic arch aneurysms. Materials and Methods. This retrospective study included 68 male and female patients with a confirmed diagnosis of either an aortic arch aneurysm or a descending thoracic aortic aneurysm with a short proximal landing zone (<1.5 cm) who underwent either hybrid or endovascular treatment at the Department of Vascular Surgery between January 2017 and December 2024. Study outcomes included a composite measure of technical success, a composite measure of in-hospital clinical success, and a composite measure of long-term treatment outcomes, including stroke, myocardial infarction, and aortic-related mortality. Results. All 68 patients were divided into two groups: Group I comprised patients who underwent endovascular treatment, whereas Group II included patients who underwent hybrid surgical treatment. The groups were comparable with regard to demographic and anatomical characteristics, clinical presentation, and comorbidities. The composite technical success rate (defined as successful target stent-graft deployment without conversion to open surgery and absence of type I or type III endoleaks) was comparable between the groups at the intraoperative stage (*p* = 1.000). The composite measure of in-hospital clinical success was achieved in 33 patients (94%) in Group I and 22 patients (67%) in Group II and was significantly higher in the endovascular group (adjusted *p* = 0.005). This difference was primarily attributable to a higher incidence of complications in the hybrid treatment group, including stroke (9%) and peripheral nerve injury (9%), associated with the open surgical component of the procedure. The mean follow-up duration was shorter in Group I (19.3 ± 10.4 months) than in Group II (63.9 ± 29.5 months), reflecting the fact that most patients in Group I underwent treatment during the later years of the study period. Although a difference in the composite long-term outcome measure was observed before adjustment (*p* = 0.031), this finding did not remain statistically significant after correction for multiple testing (adjusted *p* = 1.000). Conclusions. In this preliminary single-centre study, endovascular and hybrid approaches showed comparable technical efficacy in the early postoperative period. However, hybrid surgical treatment was associated with a less favourable safety profile during the early postoperative period, as reflected by the significantly lower in-hospital composite clinical success rate and longer hospital stay than in the endovascular group. These findings remained robust after correction for multiple testing. Long-term results should be interpreted with caution and require confirmation in larger prospective studies with longer and balanced follow-up periods.

## 1. Introduction

Aortic arch pathology is being diagnosed with increasing frequency in the ageing population. Open surgical repair remains the gold standard; however, mortality and neurological complication rates remain considerable, reaching 3.4% in elective and 15.4% in emergency settings [1]. Moreover, many patients are deemed unsuitable for open surgery due to advanced age and comorbidities [2].

In recent years, dedicated branched endovascular devices, including the Castor Branched Stent-Graft, the Relay Branch Thoracic Stent-Graft System, and the NEXUS Aortic Arch Stent Graft System, have demonstrated encouraging early and mid-term clinical outcomes. However, their routine application remains limited in many countries because of restricted availability, regulatory approval, high costs, and the need for specialised institutional expertise.

Advances in endovascular technology have expanded treatment options for aortic arch disease. Nevertheless, endovascular repair of the aortic arch remains technically demanding due to its complex anatomy and the involvement of supra-aortic branches, making it one of the most challenging procedures in contemporary vascular surgery.

Several endovascular techniques are currently available. Custom-made branched and fenestrated endografts have shown favourable outcomes, but their prolonged manufacturing time and limited availability in the Russian Federation restrict routine use. The parallel graft techniques are well-established, particularly in urgent settings, though they are associated with a higher incidence of type IA endoleaks [3]. In addition, persistent “gutter” formation between the parallel graft and the main endograft has been recognised as a potential cause of delayed type IA endoleaks, aneurysm sac enlargement, and secondary interventions during long-term follow-up.

Physician-modified endografts (PMEGs) have gained popularity due to their practicality. Fenestrations can be created either on the back table before implantation (“on-the-table”) or by in situ fenestration after endograft deployment, allowing aneurysm exclusion while preserving supra-aortic branch flow without the delays associated with custom device manufacturing, making them suitable even in emergency settings [4]. Recent multicentre studies have demonstrated high technical success rates with PMEGs in experienced centres, although concerns remain regarding the long-term durability of physician-modified devices and the lack of standardisation of modification techniques. The hybrid approach combines open surgical debranching with subsequent thoracic endovascular aortic repair (TEVAR) to reduce perioperative morbidity [5]. Hybrid repair has become an important treatment option for patients considered unsuitable for conventional total arch replacement; however, it remains associated with the potential risks of supra-aortic debranching procedures and staged surgical interventions.

While short- and mid-term outcomes for both hybrid and total endovascular approaches are encouraging, evidence on long-term durability remains limited. Furthermore, most available studies are retrospective, single-centre series with heterogeneous patient populations and treatment strategies, making direct comparison between techniques challenging. Direct comparative studies are scarce, underscoring the clinical relevance of our investigation. Therefore, further comparative clinical studies are required to better define the relative safety, efficacy, and durability of contemporary endovascular and hybrid approaches in routine clinical practice.

### Study Objective

The aim of this study was to evaluate the efficacy and safety of endovascular and hybrid treatment strategies in patients with aortic arch aneurysms.

## 2. Materials and Methods

### 2.1. Study Design and Patient Population

This was a single-centre retrospective study that included 68 male and female patients with a confirmed diagnosis of either an aortic arch aneurysm or a descending thoracic aortic aneurysm with a short proximal landing zone (<1.5 cm). All patients underwent either hybrid repair or endovascular treatment at the Department of Vascular Surgery between January 2017 and December 2024.

All patients met established indications for surgical intervention based on computed tomography angiography (CTA) findings.

Treatment allocation was not randomised. The choice between total endovascular and hybrid repair was based on a multidisciplinary assessment of anatomical suitability, operative risk, patient comorbidities, vascular access, availability of endovascular devices, and the anticipated feasibility of obtaining an adequate proximal landing zone. Because dedicated branched arch devices were not routinely available during the study period, physician-modified endografts and hybrid debranching techniques represented the principal treatment strategies in our institution.

Inclusion Criteria

Patients were eligible for inclusion if they met all of the following criteria:

- Isolated aortic arch aneurysm or descending thoracic aortic aneurysm with a short proximal landing zone (<1.5 cm) meeting accepted indications for surgical repair, including:
  ○ fusiform aneurysm with a maximum diameter >55 mm on contrast-enhanced computed tomography angiography;
  ○ or saccular aneurysm of any size;
  ○ or symptomatic aneurysm, including symptoms attributable to local compressive effects.
- Common femoral artery diameter > 7 mm;
- Age ≥ 18 years.

Exclusion Criteria

Patients were excluded if any of the following conditions were present:

- Stanford type A aortic dissection or DeBakey type I–III aortic dissection;
- Severe aortic valve regurgitation or aortic stenosis;
- Hypersensitivity to iodinated contrast agents;
- Ascending aortic aneurysm;
- Thoracoabdominal aortic aneurysm or thoracoabdominal aortic dissection;
- Emergency or delayed surgical treatment for ruptured thoracic aortic aneurysm;
- Aortic arch endovascular repair combined with alternative endovascular techniques for supra-aortic branch preservation (e.g., chimney or parallel graft techniques);
- Aortic arch endovascular repair performed using a combination of hybrid and endovascular branch-revascularisation techniques (e.g., extra-anatomical bypass combined with intraoperative fenestration);
- Endograft implantation with a proximal landing zone in Ishimaru zone 0;
- Acute infectious disease or chronic infection during an active exacerbation phase;
- Documented history of hereditary connective tissue disorders;
- Bilateral aorto-femoral occlusive disease.

### 2.2. Study Groups

Patients were divided into two treatment groups:

Group I (Endovascular Group): patients who underwent total endovascular aortic arch repair using intraoperative stent-graft fenestration techniques.

Group II (Hybrid Group): patients who underwent hybrid aortic arch repair, consisting of supra-aortic vessel debranching followed by thoracic endovascular aortic repair (TEVAR).

All cases were discussed by a multidisciplinary team including a cardiac surgeon, who recommended against open repair in all patients. The final decision on the treatment strategy was made by the same leading operating surgeon, proficient in both open and endovascular techniques, who performed all procedures throughout the study period.

Throughout the study period, all procedures were performed or directly supervised by the same senior vascular surgeon with extensive experience in both hybrid and total endovascular aortic arch repair. This approach minimised inter-operator variability and ensured consistent patient selection and procedural planning during the study period.

**Figure 1:**
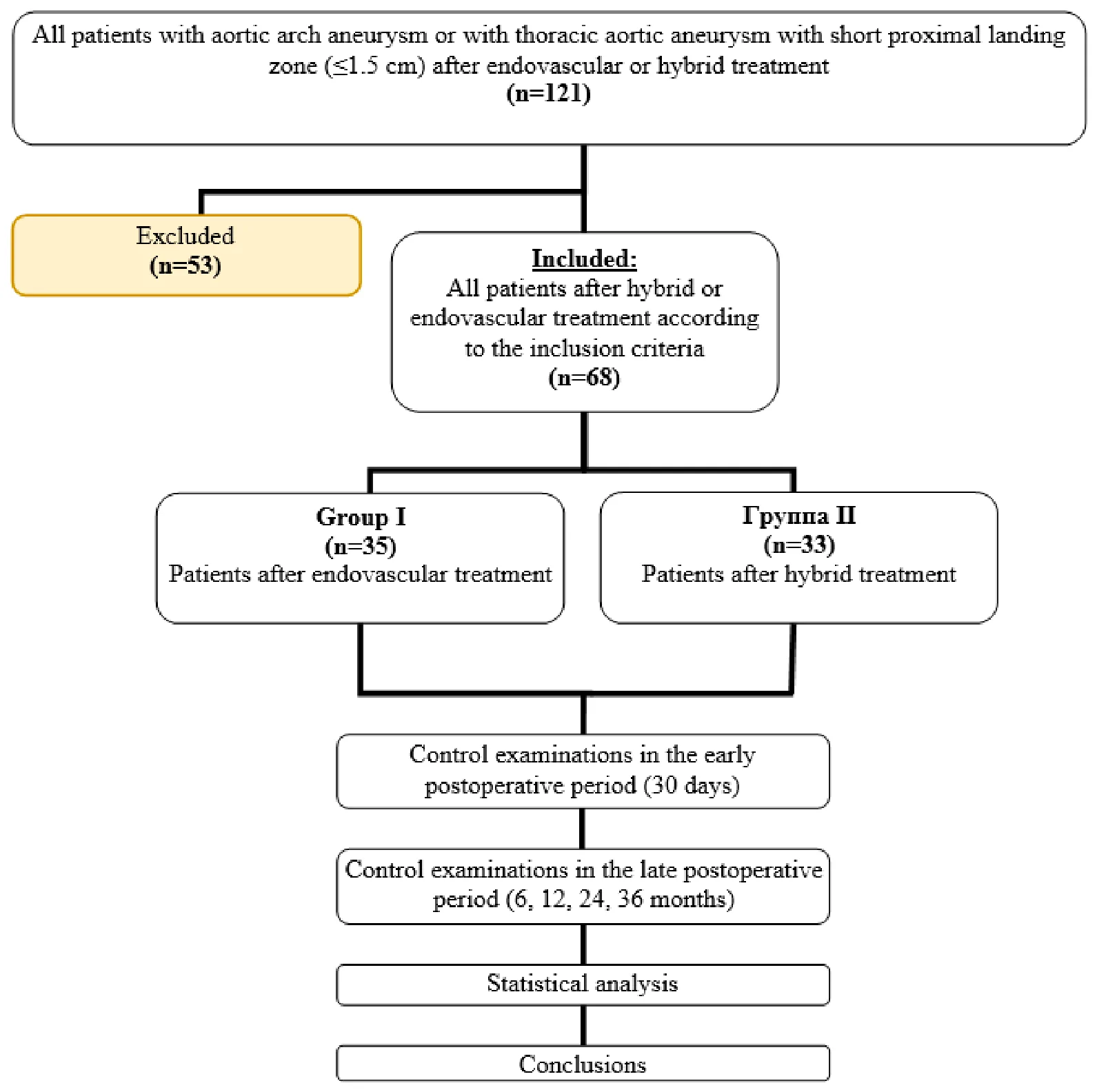
Study design.

The overall study design and patient allocation are presented in Figure 1.

All patients underwent a standard preoperative evaluation, including routine laboratory testing and cardiovascular imaging. In addition, contrast-enhanced computed tomography angiography of the aorta with a slice thickness of less than 2 mm was performed in all cases. Morphometric analysis of the aorta was subsequently undertaken to determine the optimal treatment strategy.

The following anatomical and morphological characteristics were assessed on CTA:

- Anatomy of the supra-aortic vessels;
- Length and diameter of the proximal and distal landing zones;
- Presence of angulation and morphology of the proximal landing zone;
- Aneurysm morphology and maximum aneurysm diameter.

Degree of calcification and mural thrombus formation within the proximal and distal fixation zones.

Morphometric measurements were independently reviewed by two experienced vascular surgeons using dedicated three-dimensional workstation software. Whenever discrepancies were identified, the final measurements were established by consensus during the multidisciplinary conference.

- Duplex ultrasound examination of the lower extremity arteries was performed to evaluate:
- Diameter of the common femoral arteries;
- Presence and extent of calcification at the planned vascular access site.

Characteristics of the Performed Procedures

In the hybrid group (n = 33), all patients underwent supra-aortic debranching followed by TEVAR. In the endovascular group (n = 35), all patients underwent total endovascular repair with preservation of supra-aortic branches using intraoperative stent-graft fenestration (“in situ” or “on-the-table”); thus, all devices in this group were physician-modified endografts (PMEGs). In the hybrid group, only one patient underwent a single-stage procedure; the remaining 32 patients underwent staged repair (debranching first, followed by TEVAR as a second stage).

The choice between in situ and on-the-table fenestration depended on the anatomical configuration of the supra-aortic branches, anticipated device orientation, and operator preference. In situ fenestration was generally preferred when rapid branch catheterisation could be safely achieved, whereas on-the-table fenestration was selected when preoperative planning allowed accurate alignment of the fenestration with the target vessel.

The interval between staged procedures was determined individually according to the patient’s clinical condition, recovery following supra-aortic debranching, and logistical considerations related to operating room scheduling.

For aortic stent-graft implantation, the following devices registered in the Russian Federation were used: Valiant Captivia (Medtronic, Minneapolis, MN, USA), Zenith Alpha (Cook Medical, Bloomington, IN, USA), and Ankura (Lifetech Scientific, Shenzhen, China). In the hybrid group, all three device types were used. In the endovascular group, only the Ankura stent-graft system was implanted.

Among patients in Group I, proximal stent-graft deployment in Ishimaru zone 1 was performed in 13 patients. Of these, two patients underwent a combination of on-the-table fenestration for the left common carotid artery (LCCA) and in situ fenestration for the left subclavian artery (LSA), whereas the remaining 11 patients underwent in situ fenestration of both the LCCA and LSA.

The remaining 22 patients in Group I underwent stent-graft deployment in Ishimaru zone 2. Revascularisation of the LSA was achieved by means of in situ fenestration in 21 patients, while on-the-table fenestration was used in one patient.

In situ fenestration of the aortic stent-graft was performed using a dedicated endovascular needle system via an 8F sheath placed retrogradely into the target supra-aortic branch. After puncture and advancement of a 0.018-inch guidewire into the aortic lumen, the fenestration was sequentially dilated using 5 mm and subsequently 7–8 mm balloon catheters. Finally, a covered peripheral stent-graft was implanted into the fenestrated ostium, extending 10 mm into the aortic lumen to secure branch patency and prevent endoleak.

On-the-table fenestration was performed by partially deploying the proximal 2–3 stent-graft segments from its delivery system under sterile conditions. The fenestration site was precisely marked according to preoperative CTA measurements and anatomical landmarks using radiopaque markers (the “8” and “0” markers at the 12 and 6 o’clock positions) as a reference. The fenestration was created using a scalpel or electrocautery, and a 0.018-inch guidewire tip was sutured to the fenestration edge with Prolene 6/0 to serve as a radiopaque marker for intraoperative visualisation. The device was then carefully reloaded into the delivery system, maintaining the correct rotational orientation for precise alignment with the target supra-aortic branch ostium during deployment.

In the hybrid repair group, among patients requiring proximal landing in Ishimaru zone 1, four underwent carotico–carotico–subclavian bypass using a synthetic vascular graft, whereas three patients underwent left-sided carotico–subclavian transposition combined with carotico–carotid bypass.

Among the remaining 26 patients treated with stent-graft deployment in Ishimaru zone 2, left-sided carotico–subclavian bypass was performed in 17 patients and left-sided carotico–subclavian transposition in 9 patients.

The distribution of endovascular techniques, proximal landing zones, supra-aortic branch reconstructions, and stent-graft configurations is summarised in Table 1. Detailed anatomical characteristics of the study population are presented in Table 2.

**Figure 2:**
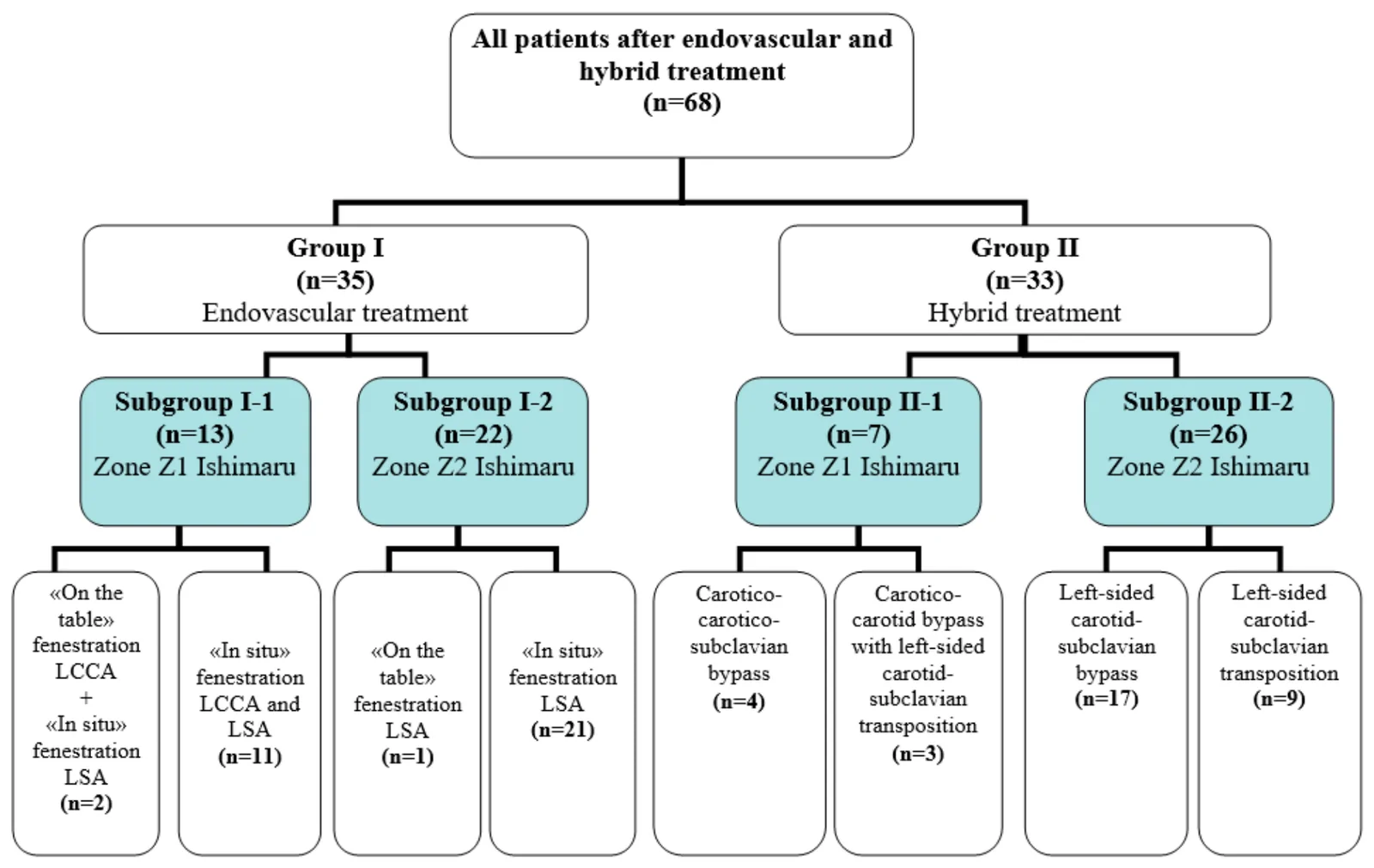
Structure of interventions in the study groups and subgroups.

The structure of the surgical procedures is presented in Figure 2.

### 2.3. Outcome Assessment

Comparative analysis of treatment outcomes was performed using intraoperative, in-hospital, and long-term follow-up data.

#### 2.3.1. Intraoperative Outcomes

The primary intraoperative endpoint was composite technical success, defined as:

- Complete exclusion of the aneurysm by the stent-graft;
- Preservation of patency of all target supra-aortic branches;
- Absence of type IA, type IB, and type III endoleaks on completion angiography;
- Successful retrieval of all delivery systems and closure of vascular access sites without conversion to open surgical repair.

#### 2.3.2. In-Hospital Outcomes

The primary in-hospital endpoint was composite clinical success, defined as technical success together with the absence of major postoperative complications during hospitalisation, including:

- Access-related vascular complications;
- Major adverse cardiovascular events (myocardial infarction and stroke);
- Peripheral nerve injury;
- Contrast-induced nephropathy.

Technical success was assessed according to the reporting standards of the Society for Vascular Surgery and the European Association for Cardio-Thoracic Surgery, based on completion angiography and operative records.

Clinical success was evaluated retrospectively using predefined objective criteria derived from electronic medical records, operative reports, discharge summaries, and postoperative imaging studies.

#### 2.3.3. Long-Term Outcomes

The primary long-term endpoint was freedom from:

- Major adverse cardiovascular events (myocardial infarction and stroke);
- Aortic-related mortality.

Prior to hospital discharge, all patients underwent contrast-enhanced computed tomography angiography of the thoracic aorta. Following discharge, CTA surveillance was recommended at 6 months and 12 months after the procedure and annually thereafter.

Whenever follow-up CTA examinations were unavailable, survival status, reinterventions, neurological events, and cardiovascular outcomes were verified using hospital records and direct patient or family contact. Patients without available imaging were included in the clinical follow-up analyses but were not considered evaluable for imaging-based endpoints such as endoleak assessment.

In addition, all patients were contacted by telephone and underwent structured follow-up interviews to assess clinical status and late adverse events.

All components of the composite endpoints were assessed retrospectively by the study investigators through review of electronic medical records, operative reports, and contrast-enhanced computed tomography angiography studies.

#### 2.3.4. Statistical Analysis

Statistical analysis was performed using RStudio IDE (version 2024.04.2+764 © 2009–2024 Posit Software, PBC, USA) with R language (version 4.1.3 (2022-03-10), Austria) and the following packages: dplyr (version 1.0.8) for data manipulation, binom (version 1.1-1.1) for binary data analysis, and ggplot2 (version 3.3.6 © Posit Software, PBC, USA) for graphics.

Since more than 30% of continuous variables deviated from a normal distribution and the sample size was small, which reduced the power of the tests, quartiles were used for descriptive statistics, and intergroup comparisons were performed using nonparametric tests.

Descriptive characteristics are presented as median [first quartile; third quartile] (MED [Q1; Q3]) for continuous data; number of patients, frequency [lower 95% CI; upper 95% CI] for binary data with confidence intervals (CIs) calculated using the Wilson method; and number and frequency of patients in each category for categorical data.

To test hypotheses regarding the equality of numerical characteristics of sample distributions between compared groups, the Mann–Whitney U test was used. The absolute shift in distributions was estimated by calculating the pseudomedian of pairwise differences between groups with 95% CIs for the shift, and the relative shift (relative to the averaged deviation) was estimated by calculating the standardised mean difference with 95% CIs. Binary and categorical variables were compared between groups using Fisher’s exact test. Comparison of complication dynamics between groups was performed by constructing Kaplan–Meier survival curves, comparing the curves using the log-rank test, and estimating hazard ratios (HRs) using the Cox proportional hazards model.

Because of the exploratory nature of this retrospective study and the limited number of outcome events, multivariable regression and propensity-score matching were not performed, as these methods would have produced statistically unstable estimates. Instead, adjusted interpretation of between-group differences was based on careful assessment of baseline characteristics and predefined study limitations.

All comparative tests were two-sided. Statistical hypothesis testing was performed at a critical significance level of *p* = 0.05, i.e., a difference was considered statistically significant if *p* < 0.05. To account for multiple comparisons, the Holm–Bonferroni correction was applied.

Kaplan–Meier analysis was used for descriptive comparison of time-to-event outcomes. Owing to the small number of deaths and reinterventions observed during follow-up, competing-risk regression (Fine–Gray model) was not performed because reliable estimation of subdistribution hazards requires a substantially larger number of outcome events.

## 3. Results

The two study groups were comparable with respect to baseline clinical and demographic characteristics (Table 1).

A predominance of male patients was observed in both groups, with no statistically significant difference in sex distribution between the endovascular and hybrid repair cohorts (63% vs. 85% male patients, respectively; *p* = 0.055). Younger age was not considered an exclusion criterion provided that no underlying connective tissue disorder was present. The mean age of patients was 66.7 ± 10.7 years in the endovascular group and 61.5 ± 11.9 years in the hybrid group, with no statistically significant difference between groups (*p* = 0.051).

Body mass index was comparable between groups (27.6 ± 5.6 kg/m^2^ vs. 27.4 ± 4.0 kg/m^2^; *p* = 0.685). Although a higher proportion of patients with obesity was observed in the hybrid repair group, this difference did not reach statistical significance (*p* = 0.053).

Similarly, no significant between-group differences were identified regarding the prevalence of major cardiovascular risk factors and comorbidities, including coronary heart disease (*p* = 0.898), hypertension (*p* = 0.886), previous myocardial infarction (*p* = 0.478), previous stroke (*p* = 0.749), chronic lower limb ischemia (*p* = 0.599), diabetes mellitus (*p* = 0.199), chronic kidney disease (*p* = 0.478), or smoking history (*p* = 0.252).

A higher prevalence of chronic obstructive pulmonary disease was observed in the endovascular group (20% vs. 3%); however, this difference did not achieve statistical significance (*p* = 0.055).

Overall, the absence of statistically significant differences in baseline demographic characteristics, cardiovascular risk factors, and comorbid conditions suggests that the two groups were well balanced and suitable for comparative outcome analysis.

We also performed a comparative analysis of aortic morphometric parameters at several levels: aortic diameter at the sinuses of Valsalva, ascending aorta, proximal and distal landing zones, and the maximum aneurysm diameter. These data are presented in more detail in Table 2. No statistically significant differences were found between the patient groups.

### 3.1. Intraoperative Outcomes

Intraoperative outcomes are summarised in Table 3.

Successful target stent-graft deployment, as assessed by completion angiography, was achieved in all patients in both groups (*p* = 1.000). No type I or type III endoleaks were detected on intraoperative angiography in either group (*p* = 1.000). Likewise, no cases required conversion to open surgical repair (*p* = 1.000).

Consequently, the composite endpoint of technical success was achieved in all patients in both groups, with no statistically significant difference observed between treatment strategies (100% vs. 100%; *p* = 1.000).

Hybrid procedures were performed as a staged intervention consisting of an initial open surgical debranching procedure followed by a subsequent endovascular repair; only in one patient were both stages performed simultaneously. The interval between the two treatment stages demonstrated a markedly skewed distribution. Therefore, it is presented as median (IQR) and range rather than mean ± standard deviation. The prolonged intervals observed in several patients reflected individual clinical circumstances, including recovery following supra-aortic debranching, optimisation of comorbid conditions, and scheduling of the second-stage procedure.

No significant difference was observed in the duration of the endovascular procedure between groups (*p* = 0.720), despite the fact that patients undergoing total endovascular repair required not only deployment of the main stent-graft body but also intraoperative fenestration and implantation of additional bridging stent-grafts for supra-aortic branch preservation.

### 3.2. In-Hospital Outcomes

In-hospital outcomes are presented in Table 4.

The composite endpoint of clinical success was achieved in 33 patients (94%) in the endovascular group and 22 patients (67%) in the hybrid group. This difference was statistically significant (*p* = 0.005).

Patients undergoing hybrid repair demonstrated a trend towards a higher incidence of postoperative stroke (9%) and peripheral nerve injury (9%), likely attributable to the open surgical component of the procedure. Of the three postoperative strokes observed in the hybrid group, one occurred following supra-aortic debranching and two occurred after the endovascular stage. Likewise, peripheral nerve injuries were attributable exclusively to the open surgical debranching procedure. This distinction should be considered when interpreting perioperative outcomes after staged hybrid repair.

Among patients who developed peripheral nerve injury, two patients (6%) sustained recurrent laryngeal nerve injury resulting in unilateral vocal cord paresis, whereas one patient (3%) experienced phrenic nerve injury leading to postoperative respiratory insufficiency.

Two patients in the hybrid group developed postoperative myocardial infarction. One patient experienced a type 1 myocardial infarction requiring urgent myocardial revascularisation by percutaneous coronary intervention. The second patient developed a type 2 myocardial infarction associated with acute decompensation of chronic heart failure, which was successfully managed conservatively.

With respect to access-related complications, one patient (3%) in the endovascular group developed brachial artery thrombosis resulting in acute ischaemia of the left upper limb and subsequently underwent emergency thrombectomy.

In the hybrid group, one patient (3%) developed access-site bleeding requiring surgical wound exploration and haemostasis, while another patient (3%) experienced postoperative lymphorrhoea, which was successfully managed with conservative wound care.

Because hybrid treatment consisted of two separate procedural stages, comparative analysis of hospitalisation parameters was limited to the endovascular stage. The mean length of hospital stay following endovascular repair was significantly shorter in the endovascular group than after the endovascular stage of hybrid repair (5.89 ± 3.47 vs. 9.94 ± 10.97 days, respectively; *p* = 0.012).

In the hybrid group, the mean postoperative hospital stay following the open surgical stage was 8.1 ± 4.01 days, whereas the mean interval between the two treatment stages was 43.38 ± 98.09 days.

No in-hospital deaths occurred in either group, resulting in a hospital mortality rate of 0% in both cohorts.

### 3.3. Long-Term Outcomes

Long-term outcomes are summarised in Table 5.

The mean follow-up duration was significantly longer in the hybrid group than in the endovascular group (63.9 ± 29.5 vs. 19.3 ± 10.4 months, respectively; *p* < 0.001).

**Figure 3:**
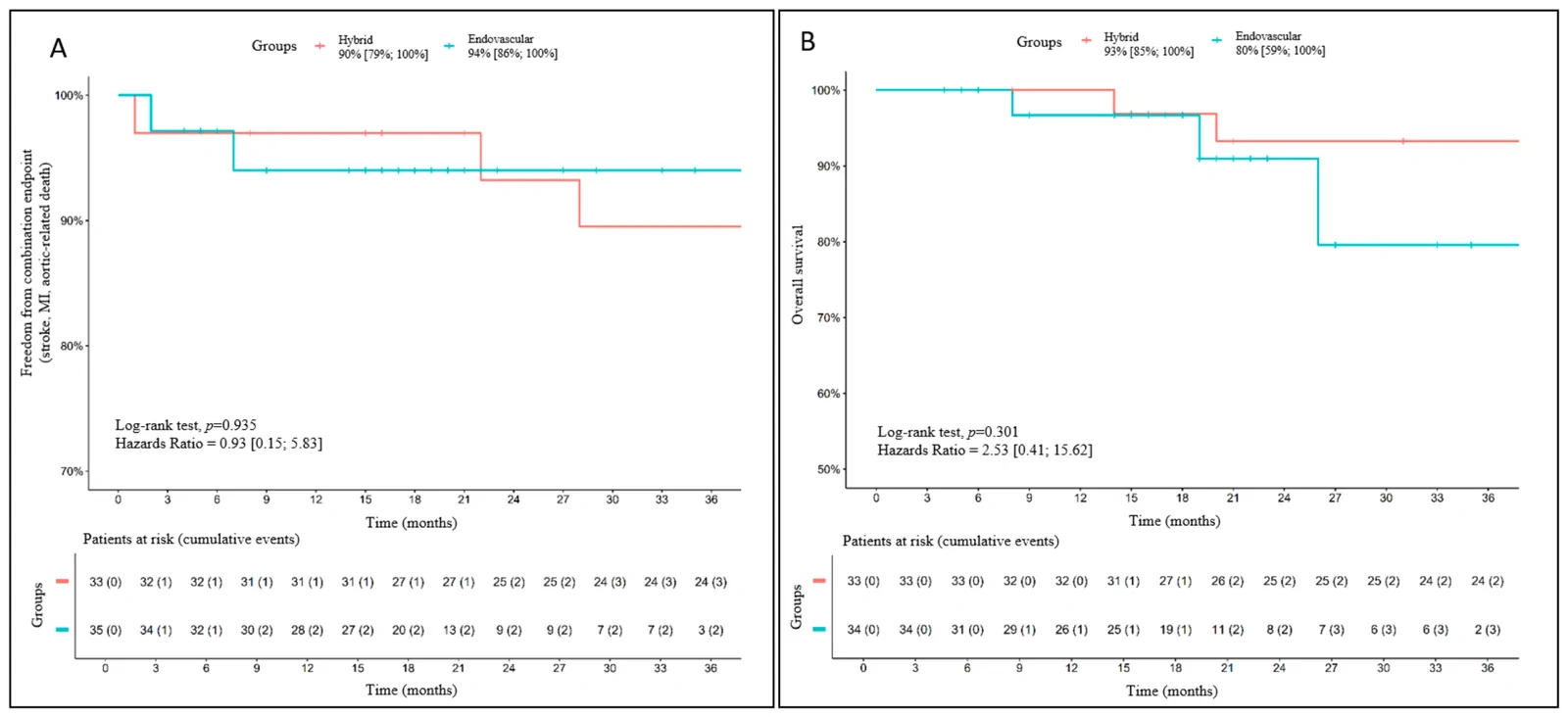
(**A**) Kaplan–Meier curve demonstrating freedom from the composite endpoint (stroke, myocardial infarction, aorta-related mortality). (**B**) Kaplan–Meier curve demonstrating overall survival.

Before adjustment for multiple testing, a difference was observed between the groups with respect to the composite long-term endpoint (freedom from stroke, myocardial infarction, aortic-related mortality; raw *p* = 0.031). However, after applying the Holm–Bonferroni correction for the 60 comparisons performed, this difference no longer reached statistical significance (adjusted *p* = 1.000). The Kaplan–Meier analysis also showed no significant difference between the groups for this composite endpoint (log-rank *p* = 0.935) and survival (log-rank *p* = 0.301) (Figure 3).

Although Kaplan–Meier estimates at 36 months suggested numerically higher freedom from the composite endpoint after total endovascular repair, these estimates were based on a limited number of patients remaining at risk because of the substantially shorter duration of follow-up in the endovascular cohort. Therefore, these findings should be regarded as descriptive only and should not be interpreted as evidence of superior long-term clinical outcomes.

During long-term follow-up, stroke occurred in two patients (6%) in each treatment group. Aortic-related mortality was observed exclusively in the hybrid repair group, affecting three patients (9%). The causes of aortic-related death included one case of thoracoabdominal aortic aneurysm rupture, one case of acute type I aortic dissection according to the DeBakey classification, and one case of rupture involving the descending thoracic aorta.

Reintervention was required in one patient (3%) in the endovascular group because of occlusion of the bridging stent-graft implanted in the left subclavian artery. In the hybrid repair group, two patients (6%) underwent reintervention owing to occlusion of the carotid–subclavian bypass graft. An additional patient (3%) required secondary thoracic endovascular aortic repair because of aneurysm enlargement distal to the implanted stent-graft associated with the development of a type IB endoleak.

No statistically significant differences were found for any individual long-term outcome after correction for multiple testing.

Because the duration of follow-up differed between the treatment groups, crude comparisons of late event rates should be interpreted with caution. Therefore, time-to-event analyses were performed to account for unequal observation periods. Kaplan–Meier curves with log-rank testing demonstrated no statistically significant differences between the endovascular and hybrid groups with respect to freedom from all-cause mortality, aortic-related mortality, or reintervention during the available follow-up period (Figure 3).

Although numerically fewer aortic-related deaths and reinterventions were observed following total endovascular repair, the limited number of outcome events resulted in wide confidence intervals and insufficient statistical power to demonstrate superiority of either treatment strategy. Accordingly, these findings should be regarded as hypothesis-generating rather than confirmatory.

Follow-up CTA beyond 24 months was unavailable in a substantial proportion of patients, particularly in the endovascular cohort. Only approximately 40% of patients in the endovascular cohort underwent CTA beyond 24 months. Consequently, the incidence of late imaging-related complications, particularly type IA endoleaks, may have been underestimated. 

Consequently, imaging-based endpoints, including the incidence of late type IA endoleaks, aneurysm sac enlargement, and delayed device-related complications, may have been underestimated. Therefore, the absence of late type IA endoleaks in the present cohort should not be interpreted as definitive evidence of long-term device durability.

Importantly, all reinterventions in the endovascular group were related to branch reconstruction rather than structural failure of the thoracic stent-graft itself. Similarly, in the hybrid cohort, most secondary procedures were associated with supra-aortic bypass graft complications, whereas only one patient required secondary TEVAR because of distal aneurysm progression with a type IB endoleak.

Overall, although no statistically significant differences were identified in baseline demographic characteristics or major comorbidities, patients in the endovascular group tended to be older and had a higher prevalence of chronic obstructive pulmonary disease. These differences most likely reflect treatment selection in routine clinical practice, whereby patients considered to be at higher operative risk were preferentially treated using a totally endovascular approach. Consequently, residual confounding cannot be excluded and should be considered when interpreting comparisons between treatment groups.

## 4. Discussion

Thoracic endovascular aortic repair has become a feasible treatment option even for patients with aortic arch pathology, with current European Society for Vascular Surgery guidelines recommending endovascular repair with proximal landing in zones 1 and 2 in suitable anatomies.

The observed tendency towards older age and a higher prevalence of chronic obstructive pulmonary disease in the endovascular cohort reflects contemporary clinical decision-making rather than differences in disease severity. In routine practice, less invasive endovascular repair is preferentially offered to patients considered poor candidates for open supra-aortic debranching because of advanced age or increased perioperative risk. Consequently, any apparent differences in clinical outcomes between the two treatment strategies should be interpreted within the context of this inherent treatment-selection bias.

Treatment allocation reflected routine clinical decision-making rather than randomisation. Patients undergoing endovascular repair were generally older and had a greater burden of comorbidities, including chronic obstructive pulmonary disease, reflecting preferential selection of less invasive treatment for individuals considered at higher operative risk. Conversely, hybrid repair was more frequently performed in patients with anatomical characteristics unsuitable for a totally endovascular approach. Consequently, residual selection bias and unmeasured confounding may have influenced both early and long-term outcomes despite broadly comparable baseline characteristics, and these findings should therefore be interpreted with appropriate caution.

Recent technological developments have substantially expanded the spectrum of endovascular treatment options for aortic arch pathology. In addition to custom-made branched and fenestrated endografts, dedicated off-the-shelf arch devices, including the NEXUS Aortic Arch Stent Graft System (Endospan, Herzliya, Israel) and the Relay Branch Thoracic Stent-Graft System (Terumo Aortic, Glasgow, United Kingdom), have demonstrated encouraging early and mid-term outcomes in selected high-risk patients. Likewise, the Castor Branched Stent-Graft has shown favourable results for proximal sealing in Ishimaru zones 1–2 in several multicentre studies. Nevertheless, the widespread use of these devices remains limited by regulatory approval, availability, anatomical eligibility, and cost, particularly in countries where customised arch devices are not routinely accessible.

To date, no published studies have directly compared endovascular and hybrid approaches for aortic arch aneurysms. Available evidence comes from systematic reviews and single- or multicentre studies evaluating each strategy separately.

Two landmark meta-analyses of hybrid arch repair (Moulakakis et al., Cao et al.) reported operative mortality rates of 10–12%, stroke rates of 6–8%, and spinal cord ischaemia in 4–7% of patients [6,7]. These findings highlight that perioperative morbidity and mortality remain substantial after hybrid repair, although patients selected for hybrid procedures often have advanced age, significant comorbidities, and elevated operative risk [8].

In our study, stroke following hybrid repair occurred in 9% (n = 3) during hospitalisation and 6% (n = 2) during follow-up. Aortic-related mortality was 9% (n = 3), and overall mortality was 12% (n = 4), consistent with published data.

While hybrid procedures reduce morbidity compared with open surgery, total endovascular repair eliminates the need for an open surgical stage [9,10]. Although custom-made branched and fenestrated endografts are unavailable in Russia, off-the-shelf devices such as the Nexus (Endospan, Herzliya, Israel) and RelayBranch (Terumo, Glasgow, United Kingdom) have emerged as promising alternatives, with favourable short- and mid-term outcomes reported in high-risk patients [11,12]. Consequently, parallel graft techniques and intraoperative fenestration remain essential tools, particularly in urgent settings. However, chimney techniques are associated with type IA endoleak rates reaching 23% due to gutter formation.

Although physician-modified endografts represent an off-label application of commercially available devices, they provide an important alternative when custom-manufactured branched grafts are unavailable or when urgent treatment is required. In the present study, all fenestrations were created either using the on-the-table modification technique or by in situ needle fenestration, both of which allowed successful preservation of supra-aortic branch perfusion with a 100% technical success rate. However, these procedures require substantial operator experience and meticulous preoperative planning, limiting their reproducibility outside high-volume aortic centres. Furthermore, the substantially shorter follow-up in the endovascular cohort precludes robust comparison of long-term durability between treatment strategies. Continued surveillance and larger prospective studies with balanced follow-up are required before conclusions regarding long-term superiority can be drawn.

Chang Shu et al. reported on 234 patients treated with chimney (n = 126), on-the-table fenestration (n = 102), and in situ fenestration (n = 6). Technical success was 99.6%, 30-day mortality 2.1%, stroke 1.7%, and type IA endoleaks occurred in 6.4% overall (11.1% in chimney vs. 1.0% in on-the-table fenestration). In our endovascular cohort, we observed zero hospital mortality, a 3% stroke rate (n = 1), and no type IA endoleaks, comparing favourably with these data [13].

The absence of type IA endoleaks in our cohort should be interpreted with caution. Long-term CTA surveillance beyond 24 months was incomplete, particularly in the endovascular group, and delayed type IA endoleaks cannot therefore be excluded. Published series have demonstrated that late gutter-related endoleaks may occur several years after chimney or parallel graft implantation, emphasising the importance of prolonged imaging surveillance.

Experience in Russia remains limited to specialised federal centres. Shlomin et al. reported 100% technical success, 10.6% hospital mortality, 87.5% 5-year survival, and 89.1% freedom from reintervention in 47 patients [14]. Imaev et al. reported on 27 patients with 100% technical success, 11% stroke, 7.4% branch occlusion, and 3.7% type II endoleak, with 30-day mortality of 7.4% [15]. These studies included heterogeneous populations and emergency procedures, limiting direct comparison with our results.

Overall, both hybrid and total endovascular approaches are viable options for aortic arch pathology, with encouraging short- and mid-term outcomes [16]. However, prospective multicentre studies and randomised trials are needed to define their comparative effectiveness and long-term durability.

When interpreting our long-term outcomes, the significant difference in follow-up duration between groups must be considered. The endovascular group had a shorter follow-up (19.3 ± 10.4 vs. 63.9 ± 29.5 months; *p* < 0.001), reflecting more recent adoption of this technique. Therefore, direct comparison of crude event rates should be interpreted with caution.

Although the endovascular group demonstrated a higher in-hospital composite clinical success rate and no cases of aortic-related mortality during the available follow-up, Kaplan–Meier analysis did not demonstrate statistically significant differences in time-to-event outcomes between treatment strategies. Furthermore, the relatively small sample size, unequal duration of follow-up, and low absolute number of adverse events resulted in wide confidence intervals, limiting the statistical power to detect clinically meaningful differences. Therefore, the present findings should be interpreted as exploratory and hypothesis-generating rather than as evidence of superiority of either treatment strategy.

## 5. Study Limitations

The findings of the present study should be interpreted in light of several important methodological limitations. This was a retrospective, single-centre analysis with a relatively limited sample size, which may restrict the generalisability of the findings. A significant difference in follow-up duration existed between the treatment groups, reflecting the more recent adoption of total endovascular techniques within our institution. The low absolute number of individual complication events (e.g., stroke, myocardial infarction, nerve injury) limits the statistical power to detect clinically relevant differences between groups for these specific endpoints. Long-term imaging surveillance was incomplete, particularly in the endovascular cohort, where computed tomography angiography at 24 months was available in only 40% of patients. Consequently, the true incidence of late endoleaks and secondary interventions may have been underestimated. Finally, the non-randomised nature of treatment allocation introduces the possibility of selection bias despite the comparable baseline characteristics observed between groups.

In addition, treatment allocation was based on multidisciplinary clinical decision-making rather than randomisation. Although baseline demographic, clinical, and anatomical characteristics were generally comparable between groups, residual confounding related to anatomical complexity, temporal changes in institutional practice, and surgeon preference cannot be completely excluded. Furthermore, because of the limited number of outcome events, propensity-score adjustment and competing-risk regression analyses were not considered statistically reliable and therefore were not performed. Finally, the present study reflects the experience of a single high-volume referral centre and should therefore be interpreted within this clinical context.

## 6. Conclusions

In this preliminary, hypothesis-generating comparative study, both total endovascular and hybrid repair achieved excellent technical success and acceptable early clinical outcomes in patients with aortic arch aneurysms. Total endovascular repair was associated with a significantly higher in-hospital composite clinical success rate and a shorter postoperative hospital stay, reflecting the avoidance of open supra-aortic debranching. However, after adjustment for multiple comparisons and time-to-event analysis, no statistically significant differences were observed between treatment strategies with respect to long-term mortality or reintervention. Consequently, the available data do not support conclusions regarding the long-term superiority of either approach. The observed numerical differences in late outcomes most likely reflect differences in patient selection and duration of follow-up rather than proven superiority of one treatment strategy over the other.

## Figures and Tables

**Table 1:** Baseline anatomical characteristics of patients.

Parameter	Group I Endovascular Repair, n = 35	Group II Hybrid Repair, n = 33	*p*-Value
Aortic diameter at the level of the sinuses of Valsalva, mm MED [Q1; Q3]	35.00 [32.00; 37.50]	38.00 [34.00; 43.00]	0.069
Ascending aortic diameter, mm MED [Q1; Q3]	33.00 [29.75; 36.25]	34.00 [31.00; 37.00]	0.502
Diameter at the level of the proximal landing zone, mm MED [Q1; Q3]	31.00 [28.00; 37.00]	34.00 [28.00; 37.00]	0.354
Diameter at the level of the distal landing zone, mm MED [Q1; Q3]	30.50 [26.75; 36.00]	32.00 [28.00; 37.00]	0.404
Maximum aneurysm diameter, mm MED [Q1; Q3]	57.00 [42.50; 64.50]	57.00 [52.00; 71.00]	0.160
Aneurysm morphology (0—fusiform; 1—saccular; 2—post-traumatic), n (%)	0–14 (40%) 1–18 (51%) 2–3 (9%)	0–22 (67%) 1–10 (30%) 2–1 (3%)	0.106

Abbreviations: MED [Q1; Q3], median [first quartile; third quartile].

**Table 2:** Clinical and demographic characteristics of the patients.

Variable	Group I Endovascular Repair, n = 35	Group II Hybrid Repair, n = 33	*p*-Value (Raw)
Age, MED [Q1; Q3]	69.00 [61.50; 73.00]	65.00 [57.00; 69.00]	0.051
Male, n (%)	22 (63%)	28 (85%)	0.055
BMI, MED [Q1; Q3]	26.80 [24.25; 29.25]	27.17 [24.50; 30.49]	0.685
Obesity, n (%)	22 (63%)	24 (72,7%)	0.053
Coronary heart disease, n (%)	3 (9%)	3 (9%)	0.898
Hypertension, n (%)	33 (94.3%)	30 (91%)	0.886
Previous myocardial infarction, n (%)	6 (17%)	3 (9%)	0.478
Previous stroke, n (%)	5 (14%)	6 (18%)	0.749
COPD, n (%)	7 (20%)	1 (3%)	0.055
Chronic lower limb ischemia, n (%)	4 (11.4%)	1 (3%)	0.599
Diabetes mellitus (type 2), n (%)	5 (14%)	1 (3%)	0.199
Chronic kidney disease, n (%)	1 (3%)	2 (6%)	0.478
Smoking history, n (%)	19 (54.3%)	13 (39%)	0.252

Abbreviations: MED [Q1; Q3], median [first quartile; third quartile]; BMI, body mass index; COPD, chronic obstructive pulmonary disease.

**Table 3:** Intraoperative outcomes.

Variable	Group I Endovascular Repair, n = 35	Group II Hybrid Repair, n = 33	*p*-Value
Composite technical success, n (%)	35 (100%)	33 (100%)	1.000
Successful target stent-graft deployment, n (%)	35 (100)	33 (100%)	1.000
Patency of all supra-aortic branches, n (%)	35 (100%)	33 (100%)	1.000
Type IA, IB, or III endoleak, n (%)	0 (0%)	0 (0%)	1.000
Conversion to open surgery, n (%)	0 (0%)	0 (0%)	1.000
Duration of open surgical stage, mean ± SD (range)	Not applicable	177.97 ± 66.02 (105–340)	–
Duration of endovascular stage, mean ± SD (range)	159.43 ± 85.91 (65–545)	146.69 ± 52.81 (80–310)	0.720

Abbreviations: SD, standard deviation.

**Table 4:** In-hospital outcomes.

Outcome Measure	Group I Endovascular Repair, n = 35	Group II Hybrid Repair, n = 33	*p*-Value	*p*-Value (Adjusted)
Composite clinical success endpoint, n (%)	33 (94%)	22 (67%)	0.005 *	0.005 *
Stroke, n (%)	1 (3%)	3 (9%)	0.331	1.000
Peripheral nerve injury, n (%)	0 (0%)	3 (9%)	0.095	1.000
Myocardial infarction, n (%)	0 (0%)	2 (6%)	0.212	1.000
Contrast-induced nephropathy, n (%)	0 (0%)	1 (3%)	0.465	1.000
Access-site vascular complications, n (%)	1 (3%)	2 (6%)	>0.999	1.000
Length of hospital stay for the open surgical stage, days, mean ± SD (range)	N/A	8.1 ± 4.01 (2.00–38.00)	—	
Length of hospital stay for the endovascular procedure, days, mean ± SD (range)	5.89 ± 3.47 (3.00–20.00)	9.94 ± 10.97 (0.00–50.00)	0.012 *	0.036 *
In-hospital mortality, n (%)	0 (0%)	0 (0%)	1.000	1.000

Abbreviations: SD, standard deviation; N/A, not applicable. * Statistically significant difference (*p* < 0.05).

**Table 5:** Long-term results.

Indicator	Group I Endovascular Repair, n = 35	Group II Hybrid Repair, n = 33	*p*-Value	*p*-Value (Adjusted)
Mean follow-up period, months; mean ± SD	19.3 ± 10.4	63.9 ± 29.5	<0.001 *	<0.001 *
Combined clinical success rate, n (%)	32 (97%)	23 (79%)	0.031 *	1.000
Cerebrovascular Accident (CVA), n (%)	2 (6%)	2 (6%)	0.493	1.000
Myocardial Infarction (MI), n (%)	0 (0%)	2 (6%)	0.239	1.000
Aorta-associated mortality, n (%)	0 (0%)	3 (9%)	0.212	1.000
Reinterventions, n (%)	1 (3%)	3 (9%)	0.614	1.000
Overall mortality, n (%)	2 (6%)	4 (12%)	0.709	1.000

* Statistically significant difference, *p* < 0.05.

## Data Availability

The original contributions presented in this study are included in the article. Further inquiries can be directed to the corresponding author.
